# Scabies outbreaks in ten care homes for elderly people: a prospective study of clinical features, epidemiology, and treatment outcomes

**DOI:** 10.1016/S1473-3099(18)30347-5

**Published:** 2018-08

**Authors:** Jackie A Cassell, Jo Middleton, Ananth Nalabanda, Stefania Lanza, Michael G Head, Jennifer Bostock, Kirsty Hewitt, Christopher Iain Jones, Charles Darley, Simran Karir, Stephen L Walker

**Affiliations:** a Department of Primary Care and Public Health, Brighton and Sussex Medical School, Falmer, UK; b Public Health England South East, Horsham, UK; c School of Life Sciences, University of Sussex, Brighton, UK; d Faculty of Medicine and Global Health Research Institute, University of Southampton, Southampton, UK; e Division of Health and Social Care Research, King's College London, London, UK; f Faculty of Infectious and Tropical Diseases, London School of Hygiene & Tropical Medicine, London, UK

## Abstract

**Background:**

Scabies outbreaks in residential and nursing care homes for elderly people are common, subject to diagnostic delay, and hard to control. We studied clinical features, epidemiology, and outcomes of outbreaks in the UK between 2014 and 2015.

**Methods:**

We did a prospective observational study in residential care homes for elderly people in southeast England that reported scabies outbreaks to Public Health England health protection teams. An outbreak was defined as two or more cases of scabies (in either residents or staff) at a single care home. All patients who provided informed consent were included; patients with dementia were included if a personal or nominated consultee (ie, a family member or nominated staff member) endorsed participation. Dermatology-trained physicians examined residents at initial clinical visits, which were followed by two mass treatments with topical scabicide as per local health protection team guidance. Follow-up clinical visits were held 6 weeks after initial visits. Scabies was diagnosed through pre-defined case definitions as definite, probable, or possible with dermatoscopy and microscopy as appropriate.

**Findings:**

230 residents were examined in ten outbreaks between Jan 23, 2014, and April 13, 2015. Median age was 86·9 years (IQR 81·5–92·3), 174 (76%) were female, and 157 (68%) had dementia. 61 (27%) residents were diagnosed with definite, probable, or possible scabies, of whom three had crusted scabies. Physical signs differed substantially from classic presentations. 31 (51%) of the 61 people diagnosed with scabies were asymptomatic, and only 25 (41%) had burrows. Mites were visualised with dermatoscopy in seven (11%) patients, and further confirmed by microscopy in three (5%). 35 (57%) cases had signs of scabies only on areas of the body that would normally be covered. Dementia was the only risk factor for a scabies diagnosis that we identified (odds ratio 2·37 [95% CI 1·38–4·07]). At clinical follow-up, 50 people who were initially diagnosed with scabies were examined. No new cases of scabies were detected, but infestation persisted in ten people.

**Interpretation:**

Clinical presentation of scabies in elderly residents of care homes differs from classic descriptions familiar to clinicians. This difference probably contributes to delayed recognition and suboptimal management in this vulnerable group. Dermatoscopy and microscopy were of little value. Health-care workers should be aware of the different presentation of scabies in elderly people, and should do thorough examinations, particularly in people with dementia.

**Funding:**

Public Health England and British Skin Foundation.

## Introduction

**Figure 1:**
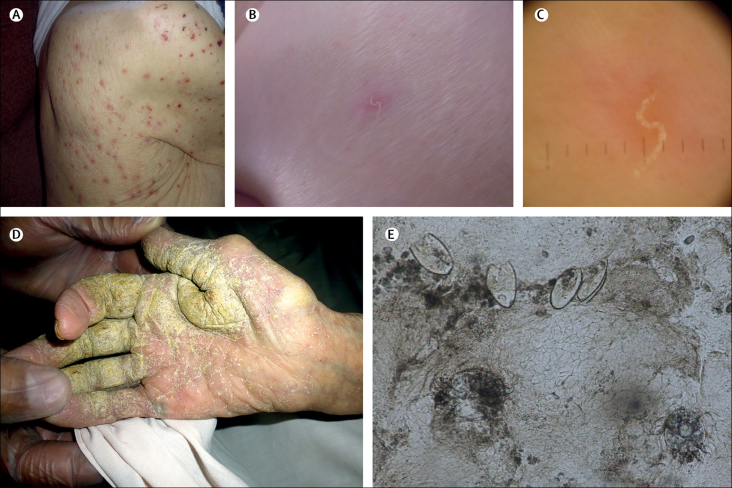
Scabies signs: papules (A), burrows (B), burrows under dermatoscopy (C), hyperkeratotic skin crusts (D), and *Sarcoptes scabiei* mites and eggs under × 10 microscopy (E)

Scabies, a skin infestation with the mite *Sarcoptes scabiei,* is often intensely pruritic and distressing. Although clinicians frequently diagnose and treat scabies in children and young adults, very old people (ie, older than 85 years) can be highly affected, and outbreaks are common in residential and nursing care for elderly people.^1^, ^2^, ^3^ The mean diagnosed incidence in the UK between 1997 and 2005 was 2·27 per 1000 men and 2·81 per 1000 women.^1^ The 2015 global burden was 5 268 900 years lived with disability.^4^ Transmission is by direct close contact and, to a lesser extent, via fomites,^5^ with a 4–6 week incubation period in people never previously infested. Signs include papules, burrows, and nodules (figure 1).^6^ Excoriation due to scratching and parasite-induced inhibition of local immune response can lead to secondary bacterial infection,^7^ with resultant risks of impaired renal function and rheumatic heart disease.^5^, ^8^ The normal parasitic burden is around 11 burrowing adult female mites per individual.^9^ However, some people develop crusted scabies, with hyperkeratotic skin lesions (figure 1) harbouring more than 4700 mites per g.^10^ Such patients have an increased mortality risk and are core transmitters during outbreaks.^10^, ^11^

Scabies outbreaks in residential care homes for elderly people are challenging to diagnose, stigmatising, and costly to manage.^11^ The close proximity of individuals and lengthy asymptomatic incubation period enables many residents and staff to become infested before detection, which is often delayed.^3^ Management of outbreaks comprises repeated simultaneous mass treatments with topical scabicide applied over the whole body and environmental decontamination.^12^ Mass treatments are logistically challenging, labour intensive, and can distress residents, many of whom have dementia and might not understand why they are receiving treatment.^3^ In a retrospective review^11^ of institutional scabies outbreaks (mainly in residential care homes for elderly people and hospitals), Mounsey and colleagues reported a median attack rate of 38% and that outbreaks usually persisted for many months, with cases of crusted scabies being the index in 83%.

**Research in context**

**Evidence before this study**

This prospective study followed on from a needs assessment and a retrospective interview-based study of scabies outbreaks in residential care homes in southeast England done by some of the authors. For that needs assessment, PubMed was searched with the terms “scabies”, “care homes”, and “residential homes”, and Google Scholar was searched with the term “scabies care homes”, to identify articles in English published before Sept 11, 2010. The reference lists of articles identified by that search were also screened for inclusion. Eight case studies, six review articles, and five epidemiological studies were identified and included. The search showed that scabies outbreaks in residential and nursing care homes for elderly people are little studied, with no research on the topic in the UK before our work. However, the data and literature examined in the needs assessment, alongside the retrospective study, suggested that outbreaks are common in residential care homes, diagnosis is often delayed leading to avoidable transmission and distress, and clinical appearance of scabies in elderly populations was not what clinicians expected. We did a follow-up search of PubMed, Cinahl, Embase, and Web of Science for articles published in English up to July 19, 2017 (the search string is in the appendix, p 7). We also used Google Scholar citation searching. Two relevant case reports were identified by this search, but neither included additional themes or clinical data to those shown by the needs assessment and retrospective study.

**Added value of this study**

In this study of ten scabies outbreaks in residential care homes for elderly people, we assessed risk factors, outbreak characteristics, and clinical presentation. To our knowledge, it is the largest prospective clinical study so far on this topic. We described how scabies presentation in this elderly population differed from classic descriptions, which are based on younger age groups. For example, the hands are a very common location for burrows in younger people, yet burrows were noted in only a minority of our elderly cohort. Furthermore, more than half of participants had physical signs only at sites normally covered by clothing, and more than half of those diagnosed were asymptomatic. We also showed that dementia is a risk factor for scabies in residential care homes, and described outbreak characteristics. The distinctive clinical characteristics in elderly populations, combined with the limited value of diagnostic tools, could contribute to delayed diagnosis.

**Implications of all the available evidence**

Scabies outbreaks are a common public health problem in residential care homes. To achieve timely outbreak detection and minimise transmission and distress in this vulnerable group, clinicians should be educated about the presentation of scabies in care homes, and novel diagnostics need to be developed. Topical mass treatments are distressing and labour intensive, so use of oral drugs should be considered in countries where they are not presently licensed for scabies.

Previous work has suggested that clinical presentation in elderly people might differ from that in younger age groups,^13^ and that diagnostic delay could contribute to outbreak size.^3^ However, no prospective studies so far have investigated clinical presentation in several outbreaks with clear case definitions in the context of the host community. We hypothesised that differing clinical presentation in elderly people could contribute to delayed outbreak detection and suboptimal management. We aimed to establish the clinical characteristics of scabies in a population comprising elderly residents of UK care homes during an outbreak, resident characteristics predictive of scabies diagnosis, outbreak characteristics, and treatment effectiveness. In particular, we sought to characterise the distribution and duration of signs and symptoms and the course of outbreaks in this population.

## Methods

### Study design and participants

We did a prospective observational study between Jan 23, 2014, and April 13, 2015, in residential care homes for elderly people in southeast England that reported scabies outbreaks to Public Health England health protection teams. An outbreak was defined as two or more cases of scabies (in either residents or staff) at a single care home. All outbreaks were eligible for inclusion. Our study was approved by the Camberwell St Giles Research Ethics Committee. Informed consent was sought from all participants. Research staff did capacity assessments of residents with known cognitive impairment. If residents lacked capacity to consent, advice was sought from personal consultees (normally relatives) or nominated consultees (care home staff) as described elsewhere.^14^ The protocol is available online.

### Procedures

Preliminary visits to each care home were done to gather data about outbreak characteristics, assess residents' mental capacity, and recruit participants. Residents were examined at initial clinical visits, which were followed by two mass treatments with topical scabicide according to local health protection team guidance. Follow-up clinical visits were arranged for approximately 6 weeks after initial visits, because symptoms can persist for up to 6 weeks after effective treatment,^15^ and experts suggest such symptoms should be investigated 4 weeks after treatment.^16^ Capacity was reassessed at both clinical visits before examination. We used a prioritisation strategy for examinations (appendix p 1).

Data for age, mentia diagnosis (as recorded in care-home records), continence, mobility, medical history, and medication use were collected at preliminary visits and initial clinical visits. Characteristics of homes and outbreaks that were recorded included demographics, number and proportion of residents affected, number and proportion treated, time to diagnosis, time to treatment, type of ownership, classification (with or without nursing), number of residents and maximum capacity, number of sections or floors, how the outbreak was detected, and number of staff affected. Scabies diagnostic criteria were developed (panel), and cases of crusted scabies were graded on the basis of the clinical scale devised by Davis and colleagues.^17^ Morphology and location of signs were recorded, with locations grouped into areas normally covered or uncovered (appendix p 2).

Panel **Scabies diagnostic criteria**

**Definite**

Clinical features suggestive of scabies (ie, primary or secondary lesions*) plus a visible mite under dermatoscopy (positive delta wing sign) or a skin scraping or biopsy with identified mites, mite eggs, or faeces

**Probable**

Clinical features suggestive of scabies (ie, primary or secondary lesions*) at common sites of scabies infestation but no mite or mite products identified by dermatoscopy, skin scraping, or biopsy

**Possible**

Non-specific rash (which can include papules, vesicles, or pustules) at sites that are uncommon for scabies infestation; a positive family or social contact history with a definite case of scabies

**Crusted**

Clinical symptoms suggestive of scabies, hyperkeratotic skin crusts and skin fissuring as a result of hyperinfestation

When possible, examinations were done by two clinicians working together (at 13 of 20 visits), who used dermatoscopes (Heine Delta 20 Plus; Heine Optotechnik, Herrsching, Germany) and took clinical photographs as appropriate. The clinical team consisted of two consultant dermatologists and two primary care physicians with dermatology certification, and the same clinicians attended initial and follow-up visits (appendix p 3). Skin scrapes from participants with definite or probable scabies were examined under microscopy by senior specialist biomedical scientists the day after the sample was taken. Demographic and medical data were collected by consulting care-home staff and records (resident files, medication sheets, reports of clinician visits, hospital discharge letters, and ambulance-service assessments), usually at initial clinical visits. Time to diagnosis (in days) for individual residents with scabies was defined as the time between first awareness of signs or symptoms (earliest report by resident or staff, or earliest recording in care-home records) to date of diagnosis. We used a structured questionnaire to interview managers about home and outbreak characteristics. Death certificates were obtained from the UK General Register Office for all participants who died before follow-up. To reduce potential for bias at follow-up, study clinicians were not reminded of diagnoses made at initial visits.

### Statistical analysis

Study size was based on the number of outbreaks reported to health protection teams that agreed to participate and could be visited before mass treatment within the funding period. A model was developed to predict diagnosis of scabies at initial clinical visit. Variables for the model were pre-specified to represent plausible causal pathways to an increased risk of scabies. We followed the rule of ten events per variable, because too many variables in the model would potentially result in overfitting. Data-reduction methods were used on the candidate list to reduce risk of multicollinearity, missing data, and sparsely populated categories (appendix p 2). We included a random effect to account for resident clustering within care homes. Because of the small number of clusters, normal distributions of test statistics would not have been a reasonable assumption, and so estimates were bootstrapped, and random samples were drawn with replacement. Adjusted odds ratios were calculated by fitting a logistic mixed-effects model for scabies diagnosis as a binary variable (no sign of scabies or diagnosis of scabies) with xtmelogit, with care home included as a random effect. Statistical analysis was done in Stata (version 14.1).

### Role of the funding source

The study funders had no role in study design; data collection, analysis, or interpretation; or writing of the report. However, JAC and KH had honorary or substantive employment contracts with Public Health England (or its predecessor, the Health Protection Agency). The corresponding author had full access to all the data in the study and had final responsibility for the decision to submit for publication.

## Results

33 scabies outbreaks were reported to the study. Seven care homes did not wish to take part, and we could not schedule visits without delaying mass treatment at a further 16. Thus, ten homes with 430 residents were visited. Preliminary visits to homes took place a median of 2 days (IQR 1–7) after health protection teams reported outbreaks to us. Initial clinic visits were done a median of 5 days (IQR 3–8, not including one pilot visit done 5 days after initial treatment) after notification of outbreak.

**Figure 2:**
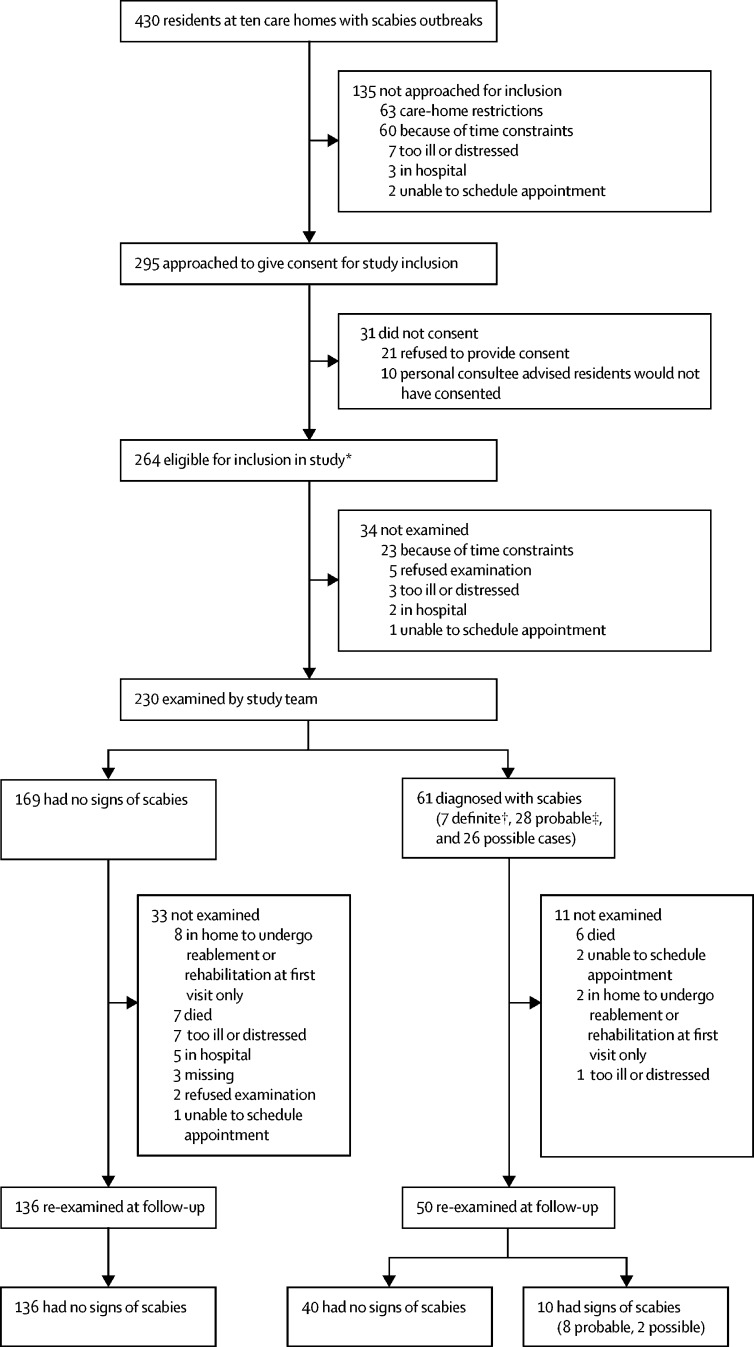
Participant profile 23 residents received only partial examinations at initial visits: 14 were distressed, two were in pain, one was sick, one refused examination, and five for other reasons. At follow-up, 16 received partial examinations: ten were distressed, two were in pain, one was sick, two had insufficient mobility, and one for another reason. *116 residents consented personally, whereas for 124 residents a personal consultee advised they would have wanted to participate had they had capacity; in a further 24 residents, a nominated consultee gave positive advice. †Includes two residents with crusted scabies, one of whom (with grade 2 crusted scabies) died 4 days after the initial visit. ‡Includes one resident with crusted scabies. Further data about clinical progression of examined residents at follow-up is included in the appendix (p8).

135 of these residents were not approached for inclusion (figure 2). The mental capacity of the remaining 295 residents was assessed. 144 (49%) had capacity to consent, 151 (51%) did not). 230—who either consented or, if they were unable to do so, had a designated consultee endorse participation on their behalf—were examined at initial clinical visits (figure 2). 186 (81%) patients were examined at follow up (figure 2). Median age was 86·9 years (IQR 81·5–92·3), and the age distribution of participants was similar to national estimates of age at admission to residential and nursing care homes (appendix p 7).^18^ 174 residents (76%) were female, and 157 (68%) had dementia (table 1). The extent of polypharmacy and comorbidity did not differ noticeably between patients with scabies and those without scabies (data not shown).

**Table 1:** Resident demographics and scabies diagnoses

	**Overall (n=230)**	**Initial scabies diagnosis**	
		No sign of scabies (n=169)	Possible, probable, or definite scabies (n=61)
**Sex**			
Female	174 (76%)	130 (77%)	44 (72%)
Male	56 (24%)	39 (23%)	17 (28%)
**Age group, years**			
<70	14 (6%)	9 (5%)	5 (8%)
70–74	8 (3%)	6 (4%)	2 (3%)
75–79	29 (13%)	20 (12%)	9 (15%)
80–84	35 (15%)	28 (17%)	7 (11%)
85–89	62 (27%)	46 (27%)	16 (26%)
90–94	55 (24%)	37 (22%)	18 (30%)
95–99	23 (10%)	19 (11%)	4 (7%)
>100	4 (2%)	4 (2%)	0
Median (IQR)	86·9 (81·5–92·3)	87·0 (81·7–92·5)	86·4 (79·8–91·9)
**Dementia**			
No	73 (32%)	58 (34%)	15 (25%)
Yes	157 (68%)	111 (66%)	46 (75%)
**Continence**			
Continent	80 (35%)	59 (35%)	21 (34%)
Urinary incontinence	44 (19%)	33 (20%)	11 (18%)
Faecal incontinence	5 (2%)	4 (2%)	1 (2%)
Urinary and faecal incontinence	101 (44%)	73 (43%)	28 (46%)
**Mobility**			
Mobile	151 (66%)	108 (64%)	43 (70%)
Transfer with help	50 (22%)	35 (21%)	15 (25%)
Bedbound	22 (10%)	19 (11%)	3 (5%)
Other	7 (3%)	7 (4%)	0
**Resident type**			
Not collected	27 (12%)	18 (11%)	9 (15%)
Permanent	176 (77%)	131 (78%)	45 (74%)
Respite	16 (7%)	13 (8%)	3 (5%)
Other	11 (5%)	7 (4%)	4 (7%)
**Conditions or therapies affecting immunity**			
Cancer	31 (13%)	24 (14%)	7 (11%)
Diabetes	35 (15%)	26 (15%)	9 (15%)
Topical corticosteroids	41 (18%)	27 (16%)	14 (23%)
Systemic corticosteroids	10 (4%)	9 (5%)	1 (2%)
Nutritional problems	30 (13%)	20 (12%)	10 (16%)
Other	14 (6%)	9 (5%)	5 (8%)

Data are n (%), unless otherwise specified.

Seven of the homes were owned privately, and three were owned by local authorities. Four homes provided nursing care. At two homes, all residents were registered with one primary care practice, whereas at all the others residents had primary care physicians from several practices. Two homes had had earlier scabies outbreaks in the previous 5 years. Six were smaller than the UK mean of 40 beds, and all were located in a region in which the proportion of self-funded residents is relatively high.^19^

A median of seven (IQR 3–10; range 2–11) residents per home were diagnosed with scabies at initial clinical visits, or previously by other clinicians (appendix p 6). Four homes had at least one case of crusted scabies. At two homes, these cases were diagnosed by the study team, whereas at the other two they had already been diagnosed by other clinicians (appendix p 6). Overall, median time to diagnosis was 22 days (IQR 7·5–186; n=48; appendix p 6). In eight homes, at least one staff member was diagnosed with scabies (either by study clinicians or others). Although offered, staff were not examined at the other two homes. Scabies outbreaks reported to health protection teams were overwhelmingly located in residential and nursing care homes and attributed to transmission within that setting. The outbreaks we attended had no unusual features and were similar to those described in our previous non-clinical studies in the same region (appendix p 6).^3^

**Figure 3:**
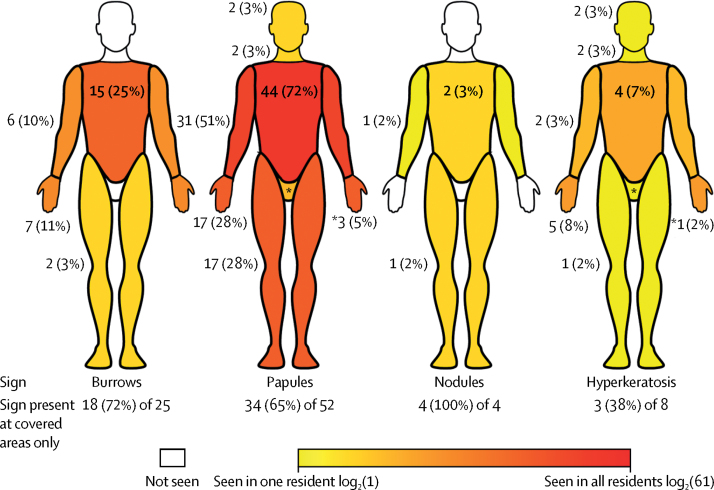
Clinical presentation of scabies in elderly care-home residents Percentages in the figure are the proportion of residents with scabies who had that sign at that location. Percentages below the figures are the number of residents with the sign only in covered locations, out of all the people with that sign. Locations that would normally be covered by clothing are the upper limbs, torso (including back), lower limbs, and genitalia. The wrist was considered to be part of the upper limb, separate from the hands. This heatmap was generated from individual patient descriptions, which provide more detail on the locations of signs (appendix pp 3–4).

At initial clinical visits, 61 (27%) residents were diagnosed with scabies: seven definite (including two crusted), 28 probable (including one crusted), and 26 possible (figure 2). Burrows were detected in 25 (41%) cases, papules in 52 (85%), hyperkeratosis in eight (13·1%), and nodules in four (7%). Figure 3 shows the distribution of signs, and resident profiles are in the appendix (pp 3–4). The torso was the most common location for burrows, hyperkeratosis, and papules (figure 3). 35 (57%) cases only had signs on normally covered areas of the body, including 18 (72%) of the 25 residents with burrows (figure 3). A scabies mite was visualised via dermatoscopy in seven infested residents (11%). Three of these residents also had positive microscopy results. Among residents not diagnosed with scabies, none had burrows, nodules, or hyperkeratosis, but five had papules (located on the torso of three residents, the hands of one, and the legs of one). Of residents with scabies diagnoses, staff reported that 31 (51%) had not complained of itch, rash, or scratching (table 2). 24 of these asymptomatic residents had dementia. Of the 16 diagnosed residents who had complained about these symptoms, only nine had complained of itch (table 2). Staff did not notice skin signs in 12 (20%) subsequently diagnosed residents. In 13 of the asymptomatic residents with scabies, signs were present only in areas normally covered by clothing.

**Table 2:** Description of skin signs and symptoms in residents before initial clinical visits as reported by care-home staff

		**Overall (n=230)**	**Scabies diagnosis at initial visit**	
			No sign of scabies (n=169)	Possible, probable, or definite scabies (n=61)
**Skin symptoms**				
Resident complained about skin-related symptoms				
	No	137 (60%)	106 (63%)	31 (51%)
	Yes	38 (17%)	22 (13%)	16 (26%)
	Unknown*	55 (24%)	41 (24%)	14 (23%)
Type (if present)				
	Itch	29 (76%)	20 (91%)	9 (56%)
	Scratching	10 (26%)	4 (18%)	6 (38%)
	Rash	13 (34%)	5 (23%)	8 (50%)
**Skin signs**				
Staff noticed skin-related signs				
	No	128 (56%)	116 (69%)	12 (20%)
	Yes	82 (36%)	35 (21%)	47 (77%)
	Unknown	20 (9%)	18 (11%)	2 (3%)
Type (if present)				
	Itch†	41 (50%)	17 (49%)	24 (51%)
	Scratching	26 (32%)	9 (26%)	17 (36%)
	Rash	46 (56%)	15 (43%)	31 (66%)

* Includes 23 residents (eight with scabies) from a pilot outbreak visit at which information about skin-related symptoms was not recorded.

† Although itch is not a sign, staff were asked whether they noticed residents who seemed itchy.

Among residents who underwent examination, dementia was significantly associated with a scabies diagnosis at the initial clinical visit (odds ratio [OR] 2·37 [95% CI 1·38–4·07]). Age (OR 0·99 [95% CI 0·93–1·04]), mobility (0·53 [0·22–1·25]), sex (1·61 [0·84–3·07]), continence (1·28 [0·53–3·11]), or conditions suppressing immunity (ie, known cancer patient, diabetes mellitus, topical steroids, on systemic steroids; 0·95 [0·51–1·76]) were not significantly associated with scabies diagnosis (appendix p 2). The intracluster correlation coefficient for residents within homes was 0·214, suggesting that clustering of data had a large effect.

In all homes, two mass treatments with topical scabicide were administered a median of 7 days apart (IQR 7–7; appendix p 5). Seven (3%) residents were not included in the first treatment, and seven (3%) were not included in the second. Because most homes obtained prescriptions from primary care physicians at more than one practice, arrangement of prescriptions and sufficient scabicide delayed treatment. Two participants living in separate homes were given oral ivermectin (appendix p 6). Staff reported that mass treatments were labour intensive, and many residents found the process distressing (data not shown). Some staff suggested oral treatment would be preferable (data not shown). In two of the four homes with cases of crusted scabies, residents with crusted scabies died before treatment completion (figure 2; appendix p 6).

No new cases of scabies were detected at follow-up visits, which were a median of 44 days (IQR 41–48) after initial clinical visits and 35 days (31–39) after second mass treatments. 50 of the 61 previously diagnosed residents were examined. 40 had no signs, and ten had either probable or possible scabies (appendix p 8). Nine of these residents had been included in the first mass treatment, and all had been included in the second. None had deteriorated, and seven had improved clinical classification (figure 2). Six (10%) of the 61 residents diagnosed with scabies died before follow-up, compared with seven (4%) of 169 of those with no signs of scabies (appendix p 5). One resident with crusted scabies (confirmed by microscopy) died in hospital 4 days after the initial clinical visit, with scabies recorded on their death certificate as a significant contributing condition (appendix p 7).

## Discussion

In our investigation of the characteristics of scabies in residential and nursing care homes, signs and symptoms of scabies differed from classic presentation. 51% of diagnosed residents were asymptomatic. By contrast with classic scabies presentation, signs were distributed largely in areas normally covered by clothing, including in participants in whom burrows were detected. A dementia diagnosis was predictive of scabies. Outbreaks were often characterised by substantial diagnostic delay, and a case of crusted scabies was detected at four care homes. Topical mass treatment was effective but distressing for residents, labour intensive, and did not result in complete resolution of signs of scabies in 20% of residents who were re-examined at follow-up.

Our study had several limitations. Scabies outbreaks attract stigma.^11^ Selection bias could thus have been introduced at recruitment, which was dependent upon care homes informing health protection teams and agreeing to participate. Most diagnoses were based on clinical signs rather than visual confirmation of mite presence. We were unable to examine all residents (figure 2; appendix p 6), and thus we cannot report attack rates. We did not access primary care or hospital records, and the accuracy of care-home records is difficult to establish. Our presence could have encouraged more thorough treatments, biasing measurement of their success.

The most common sign or symptom of scabies is itching, and the interdigital web spaces of the hands are the classic locations for signs.^15^, ^20^ However, non-classic presentation has been reported in elderly people in case reports and small studies.^13^, ^16^, ^21^ Our study also shows that presentation in elderly people in similar age ranges can differ substantially from the standard description of scabies^15^ and from clinical phenotypes, which are based primarily on paediatric populations and do not differentiate elderly populations as a distinct group.^22^ In our study, more than half of residents with scabies were asymptomatic, and many had subtle clinical signs at locations that were usually covered, which could have been missed by clinicians focusing on the sites commonly affected in younger age groups. Skin signs had gone unnoticed by staff in many cases. Burrows are pathognomonic of scabies, and hands are a typical site. In one study^23^ of male soldiers, occupied burrows were detected on the hands or wrists in 85% of 886 infested participants. By contrast, in our study, burrows were identified in only 25 (41%) patients, and were detected on the hands of only seven (12%). The extent of unexpected and hidden signs and symptoms in this population could partly explain reported delayed diagnosis. Educational materials are needed to support diagnosis of scabies in residential care homes by primary care physicians and others.

Dermatoscopy was of limited value in our study, with a mite visualised in only seven (11%) participants. In a French hospital-based study^24^ (mean participant age 33 years), dermatoscopy had 91% sensitivity, whereas in a Brazilian community-based study^25^ (median participant age 14 years), it had 83% sensitivity (appendix p 7). In the French study, 75% of cases had “involvement of typical body areas”. Our dermatoscopy findings might differ from those in previous studies because our cohort was substantially older and had different clinical features. A review^11^ by Mounsey and colleagues included 40 outbreaks in “aged care” facilities, but dermatoscopy was used in only one study (appendix p 7), and the proportion of affected individuals in whom a mite was visualised was not reported. The role of dermatoscopy in elderly people with suspected scabies warrants further study. In line with previous reports,^26^, ^27^ inspection of skin scrapings under microscopy, although useful for confirmation of positive diagnoses, was not highly sensitive. Alternative diagnostics need to be developed. In their absence, diagnosis will remain clinical. Primary care physicians might be reluctant to diagnose scabies in the absence of classic signs, especially if patients are asymptomatic, because of the implications for homes and residents.^3^ Our case definitions (panel) could be helpful, and that all but three of those examined at post-treatment follow-up had improved clinical classification lends weight to them. 19 of the 21 cases previously classified as possible were classified as no signs at post-treatment follow-up (appendix p 9). We suspect that the other two possible cases, which had not improved at follow-up, were not scabies.

To our knowledge, our study is the first to show that dementia is a risk factor for scabies in residential and nursing care homes. The explanation for this association remains unclear, but wandering behaviours and increased number of physical contacts could heighten transmission hazard.^28^ Cognitive impairment could also support infestation by reducing removal of mites through scratching.^13^ This increased risk should be considered when deciding whether to examine residents with dementia, who can have difficulty understanding and communicating symptoms. Distress caused by scabies could lead to changes in behaviour, which might mistakenly be attributed to the underlying cognitive condition rather than to the effect of scabies.

Mounsey and colleagues^11^ outlined common characteristics of institutional scabies outbreaks, many of which were evident in our study. These characteristics included protracted, delayed diagnoses (months to years in some homes in our study), residents with crusted scabies (four of the ten outbreaks in our study included at least one case), and infestation of staff (present in all homes in our study in which staff examinations took place). In agreement with some studies in that review, staff in our study reported considerable anxiety and frustration among patients and personally. Eight of the ten care homes in our study did not have a sole primary care practice responsible for all residents. Other care homes with similar arrangements could also experience impaired communication and delayed case diagnosis and outbreak detection as a result of this set-up.

Although topical scabicide treatments seemed successful, their efficacy could have been exaggerated by early exit of some participants with scabies—and particularly by the high number of deaths in the study. In half the homes with cases of crusted scabies, residents with crusted scabies died before mass treatment was finished. These patients could have been core transmitters, and, in view of the difficulty treating crusted scabies topically, their exit from the cohort could have contributed to absence of a recurrent outbreak. The distress and logistic challenges of topical mass treatments reported in this study and in previous work^3^ suggest that use of oral drugs should be considered. A suggested link between oral ivermectin treatment for scabies and excess deaths in elderly people in a much criticised 1997 study^29^ has not been reproduced.^12^ Ivermectin has an extremely good safety profile in mass drug administration programmes for filariasis and onchocerciasis.^30^ In settings where scabies prevalence is of epidemic proportions, oral ivermectin seems more effective than topical therapy.^30^ France has licensed oral ivermectin for scabies outbreaks in residential care homes, but in the UK the drug remains available only on a named patient basis and is recommended for treatment-resistant crusted scabies only.^12^ We recommend research in residential care homes comparing oral ivermectin—or novel oral treatments such as moxidectin^31^—with topical scabicides.

Care-home residents are a vulnerable population requiring advocacy. In 2011, 291 000 people aged 65 years or older lived in care homes in England and Wales,^32^ many of whom have complex health-care needs, yet the sector is arguably under-researched. Our study enabled participation in research by people with dementia. Key findings would not have been discovered had we not included such people, whose inclusion was made possible by advice on innovative methods from patient representatives. We recommend researchers consider our methods^14^ in outbreak and other time-critical studies in care homes. Ours was the first funded UK research on scabies outbreaks in care homes. The burden, effect, and management of scabies in care homes warrants further study, not least because, as the global population ages, the burden of skin disease in elderly people is expected to become a substantial challenge,^33^ and institutional disease outbreaks place substantial demands on scarce resources.

Care-home residents with scabies might be asymptomatic, and can have subtle signs at covered sites not typically affected in young people. We recommend thorough, careful examination, particularly of individuals with dementia, who are at increased risk of scabies. The need for thorough clinical examination is shown by the limited value of diagnostic tools. Scabies outbreaks are difficult to manage, distressing, and highly stigmatising. Increased awareness of how scabies presents in elderly people, alongside development of diagnostics and evidence-based treatment regimens, would help with detection and control of scabies outbreaks.

## Footnotes

* Primary lesions are burrows, papules, nodules, or vesicles; secondary lesions are excoriations due to scratching.
