# Supplementary material for: Scabies outbreaks in ten care homes for elderly people: a prospective study of clinical features, epidemiology, and treatment outcomes
Source: Lancet Infect Dis. 2018 Aug;18(8):894–902. doi: 10.1016/S1473-3099(18)30347-5 (PMC6060176; doi:10.1016/S1473-3099(18)30347-5)
Supplement: Supplementary appendix [file mmc1.pdf]

THE LANCET
Infectious Diseases

Supplementary appendix

This appendix formed part of the original submission and has been peer reviewed. We post it as supplied by the authors.

Supplement to: Cassell JA, Middleton J, Nalabanda A, et al. Scabies outbreaks in ten care homes for elderly people: a prospective study of clinical features, epidemiology, and treatment outcomes. *Lancet Infect Dis* 2018; published online June 28. http://dx.doi.org/10.1016/S1473-3099(18)30347-5.

Appendix:
Cassell JA, Middleton J, Nalabanda A. *et al.* Scabies outbreaks in ten care homes for the elderly: a prospective study of clinical features, epidemiology, and treatment outcomes

Methods in full

Study design and setting

This prospective observational study was carried out in South East England between Jan 23, 2014 and April 13, 2015, in ten homes which reported outbreaks to PHE Health Protection Teams (HPTs). An outbreak was defined as two or more cases of scabies (residents or staff) in a single RNC. Preliminary visits were undertaken to collect data on outbreak characteristics, assess residents' mental capacity, and recruit participants. Residents were examined at initial clinical visits, followed by two mass treatments with topical scabicide as per local HPT guidance. Follow up clinical visits were arranged for approximately six weeks after initial visits, as it is widely accepted symptoms may persist for up to six weeks after effective treatment,[15] and experts suggest such symptoms should be investigated after four weeks.[16]

Participants

A prioritisation strategy for examinations was used. The first priority was residents with signs or symptoms suggestive of scabies, as described by residents or staff. Secondly, residents in contact with symptomatic residents in the preceding 4–6 weeks were prioritised. In homes with <40 residents, all residents were offered examinations (six homes). In homes ≥40 residents, when outbreaks were confined to a floor or wing, all residents within affected areas were offered examinations (one home). Where outbreaks in homes with ≥40 residents were not confined to a floor or wing (two homes), as many symptomatic residents were examined as possible given time constraints, which included limited availability of RNC staff to chaperone and number of clinicians visiting. At one large home with an outbreak confined to one floor, managers insisted only residents suspected by staff were examined. Staff examinations were offered to support outbreak management, but are not reported beyond the number of homes in which affected staff were present. Research staff carried out capacity assessments of residents with known cognitive impairment. Where residents lacked capacity to consent, advice was sought from personal consultees (normally relatives) or nominated consultees (RNC staff) as described elsewhere.[14] Capacity was reassessed at both clinical visits before examination.

Data collection, processing, and statistical analysis

Data on age, sex, dementia diagnosis (as recorded in RNC records), continence, mobility, medical history, and current medication were collected. Characteristics of homes and outbreaks were collected, including demographics, number and proportion of residents affected, number and proportion treated, time to diagnosis, time to treatment, ownership, classification (with or without nursing), number of residents and maximum capacity, number of sections and/or floors, how the outbreak was detected, and number of staff affected. Scabies diagnostic criteria were developed (table 1) and cases of crusted scabies graded using the clinical scale of Davis et al.[17] Morphology and location of signs were recorded, with locations grouped into areas normally covered or uncovered (Variables and data reduction table, below).

When possible, examinations were conducted by two clinicians together (13 of 20 visits), using dermatoscopes (Heine Delta 20 Plus) and taking clinical photographs as appropriate. The clinical team consisted of two consultant dermatologists and two primary care physicians with dermatology certification, and the same clinicians attended initial and follow up visits (Examiners at clinical visits table, page 3). Skin scrapes obtained from participants with definite or probable scabies were examined next day under microscopy by senior specialist biomedical scientists. Demographic and medical data were collected by consulting RNC staff and records (resident files, medication sheets, reports of clinician visits, hospital discharge letters, and ambulance service assessments). Time to diagnosis (in days) for individual residents with scabies was defined as the time between first awareness of signs or symptoms (earliest report from resident or staff, or RNC records) to date of diagnosis. Managers were interviewed about home and outbreak characteristics using a structured questionnaire. Death certificates were obtained from the UK General Register Office for all participants who died before follow up. To reduce potential for bias at follow up, study clinicians were not informed of diagnoses given at initial visits. Study size was determined by the number of outbreaks reported to HPTs that agreed to participate and could be visited before mass treatment within the funding period.

A model was developed to predict diagnosis of scabies at initial clinical visit. Variables to go into the model were pre-specified to represent plausible causal pathways to an increased risk of scabies. We followed the rule of 10 events per variable, since too many variables in the model could have resulted in overfitting. Data reduction methods were used on the candidate list to reduce risk of multicollinearity, missing data and sparsely populated categories (Variables and data reduction table, page 2). A random effect was included to account for resident clustering within care homes. Due to the small number of clusters, normal distributions of test statistics would not have been a reasonable assumption, and so estimates were bootstrapped, drawing random samples

with replacement. A logistic mixed effects model was fitted for scabies diagnosis as a binary variable (no sign of scabies or diagnosis of scabies) using *xtmelogit*. Statistical analysis was performed in *Stata Statistical Software*: Release 14.1.

Camberwell St Giles NRES Committee approved the research, and the protocol is available at: http://sro.sussex.ac.uk/66209/.

Variable category	Model variable	Sub variable
**Potential predictors of a scabies diagnosis**		
**Age**	Continuous variable	
**Dementia**	Yes/No	
**Mobility**	Self-mobile	Walks unaided
		Walks with frame or stick
	Not self-mobile	Transfer with one helper
		Transfer with two helpers
		Immobile
		Other
**Previous treatment for scabies**	Yes/No	
**Sex**	Female/male	
**Continence**	Continent	Continent
	Continence restricted	Both urinary and faecal incontinence
		Catheter fitted
		Faecal incontinence
		Faecal incontinence and catheter fitted
		Urinary incontinence
**Immunosuppression**	Yes	Known cancer patient
		Diabetes Mellitus
		Topical steroids
		On systemic steroids
	No	None recorded
		Nutritional problems
		Other
**Covered/uncovered sites**		
**Covered sites**		Upper limbs
		Torso
		Back
		Genitalia
		Lower limbs
**Uncovered sites**		Hands
		Scalp
		Face
		Ears
		Neck

Variables and data reduction

Independent variable	Odds Ratio	p-value	Lower 95% CL	Upper 95% CL
**Age**	0.99	0.636	0.93	1.04
**Dementia (Yes vs No)**	2.37	0.002	1.38	4.07
**Mobility (Immobile vs Mobile**	0.53	0.148	0.22	1.25
**Sex (Male vs Female)**	1.61	0.149	0.84	3.07
**Continence (Incontinent vs continent)**	1.28	0.584	0.53	3.11
**Any effect on immunity (Yes vs No)**	0.95	0.867	0.51	1.76
**Constant**	0.35	0.673	0.00	43.88

Model results

Homes	Clinicians at initial examination	Clinicians at follow up examination
**A**	AN	CD, AN
**B**	SW, AN	SW, AN
**E**	CD, AN	CD, AN
**F**	CD, AN	CD, AN
**H**	CD, AN	CD, AN
**P**	MH, AN	MH, AN
**Q**	SW	SW, AN
**U**	MH, AN	AN
**V**	AN	AN
**Z**	SW	SW

Examiners at clinical visits to care homes experiencing outbreaks Examiners positions during study and years of experience in dermatology: Charles Darley (CD), consultant dermatologist, 31 years; Steve Walker (SW), consultant dermatologist, 17·5 years; Martin Heath (MH), primary care physician with dermatology certification, 15 years; Ananth Nalanbanda (AN), general practice trainee with dermatology certification.

	Burrows	Papules	Nodules	Hyperkeratosis
**AR19**	Back	Arms, Hands		
**AR7**		Scalp, Arms, Hands, Back, Legs		
**BR12**	Torso	Torso		
**BR13**		Arms, Torso		
**BR17**	Torso	Arms		
**BR18**	Arms	Arms		
**BR2**	Legs	Legs		
**BR4**	Torso			
**BR5**	Arms, Torso	Arms, Torso		
**BR6**	Back	Torso		
**ER10**		Arms, Back		
**ER12**		Arms, Back		
**ER13**		Arms, Hands, Torso, Back		
**ER16**		Arms, Hands, Back		
**ER17**		Hands, Back		
**ER18**		Arms, Hands, Torso, Back, Legs		
**ER21**		Torso, Back		
**ER3**		Arms		
**ER4**	Hands			
**ER5**	Arms, Torso	Arms, Torso		
**ER9**		Arms, Back		
**FR2**	Hands	Arms, Hands, Torso, Back		
**FR22**	Arms, Torso	Arms, Back, Legs		
**FR4**		Torso		
**FR5**		Neck, Torso		
**FR8**		Arms, Torso		
**FR9**	Torso, Back	Hands, Back		
**HR18**		Arms, Hands, Torso, Back		
**HR2**	Legs	Arms, Hands, Torso, Back, Legs		

**PR3**		Arms, Hands, Torso, Back, Legs		Arms, Hands, Torso, Back, Genitalia, Legs
**PR4**		Back		
**PR7**		Back		
**QR2**				Hands
**QR21**		Arms, Back, Legs		
**QR24**				
**QR26**		Arms, Hands, Torso, Back, Genitalia, Legs	Legs	Hands
**QR3**		Back		
**QR30**	Hands	Torso, Back, Genitalia, Legs	Torso	
**QR36**				
**QR37**	Hands, Torso	Torso, Genitalia, Legs		
**QR38**	Torso			
**QR5**	Back			
**UR12**		Back		Back
**UR20**		Back		
**UR24**			Arms, Torso	Arms
**UR3**		Back		Back
**UR6**		Torso		
**UR9**		Back, Legs		
**VR1**	Arms	Hands, Back, Legs		
**VR10**	Hands	Arms		
**VR11**		Hands, Back, Legs		
**VR2**		Arms		
**VR3**		Arms		
**VR6**		Arms, Torso		
**VR7**		Arms, Hands, Back, Legs		
**VR8**	Arms	Arms, Back		
ZR10	[Excoriations on chest and back]			
ZR2	Torso	Arms, Torso, Legs		
ZR4	Hands, Torso, Back	Scalp, Face, Ears, Neck, Arms, Hands, Torso, Back, Legs		Scalp, Face, Ears, Neck, Arms, Hands, Torso, Back
ZR6	Hands	Arms, Hands, Torso, Back, Legs		Hands
ZR9	Torso	Torso	Legs	

Profiles of signs for 61 elderly RNC residents diagnosed with scabies at initial clinical visits The wrist was not recorded as a distinct location, and was considered to be part of the upper limb separate from the hands.

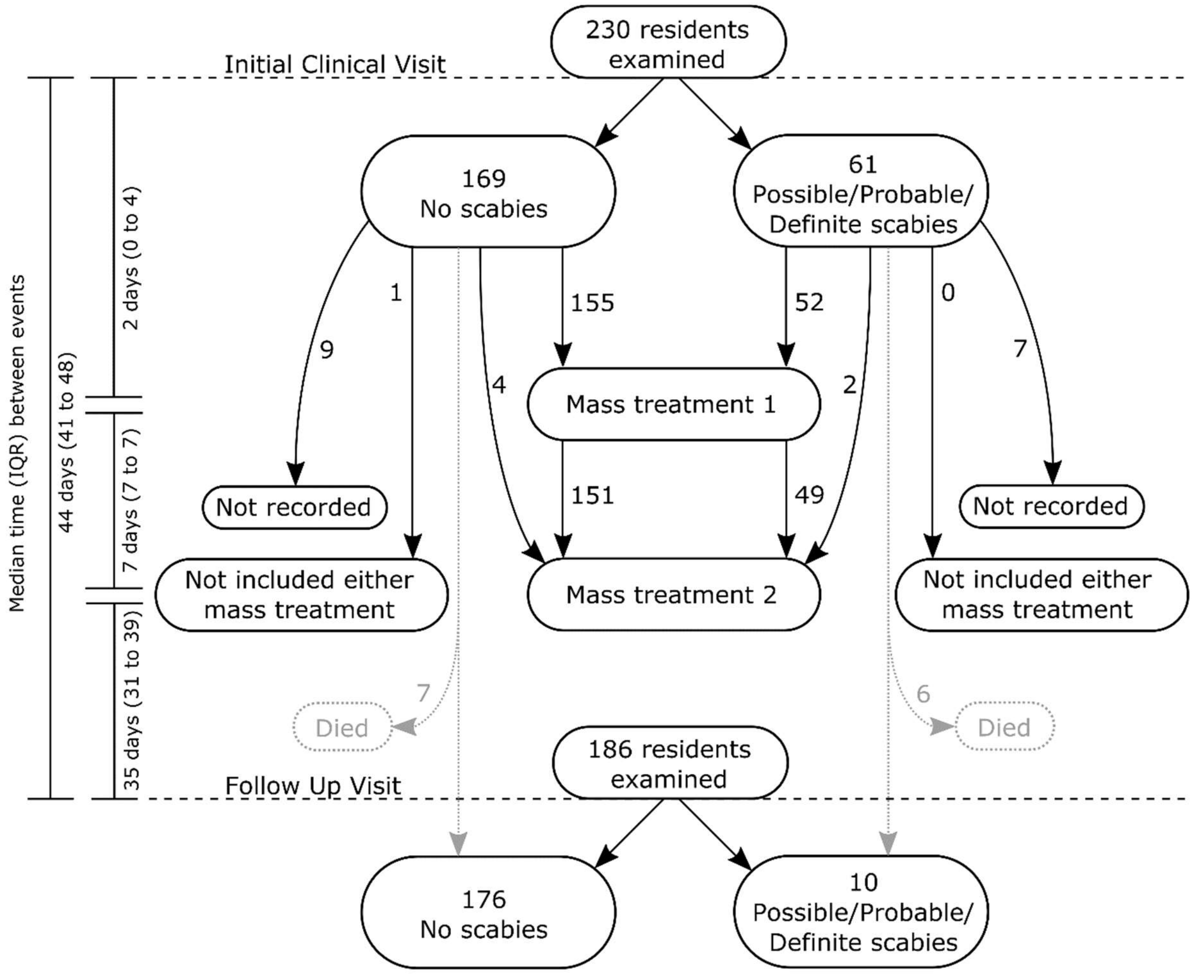

Mass treatments Mass treatments were carried out using 5% permethrin (eight homes), or a mixture of malathion and permethrin (two homes). Treatment required the scabicides remained on the entire bodies of residents and staff for 8–12 hours for permethrin, or 24 hours for malathion. Manufactures advice stated treatment should not be applied to scalp, neck, face, or ears. In line with local HPT guidance scabicide was applied to these areas, including for nine of the ten residents with signs at follow up (data missing for one). Seven residents were not included in the second mass treatment. One of these was not included in either mass treatment. The remaining six residents were included in the first but not the second, the latter is not shown diagrammatically to maintain flow chart simplicity

Care home outbreak	A	B	E	F	H	P	Q	U	V	Z	
**Classification – with nursing**		Yes	Yes		Yes					Yes	
**Resident median age (IQR)**	90.1 (83.1-95.5)	85.5 (78.2-91.2)	86.0 (82.0-91.1)	85.9 (81.4-91.9)	87.8 (82.2-91.1)	85.1 (78.0-92.6)	86.1 (79.1-92.8)	90.1 (88.2-95.1)	87.5 (77.5-90.7)	83.7 (78.4-86.6)	
**GP support**	Separate primary care physicians at various practices	Separate primary care physicians at one practice	Separate primary care physicians at various practices	Separate primary care physicians at various practices	Separate primary care physicians at various practices	Separate primary care physicians at various practices	Separate primary care physicians at various practices	Retainer with local primary care practice	Separate primary care physicians at various practices	Separate primary care physicians at various practices	
**Outbreak past 5 years**	Yes									Yes	
**Topical mass treatments**	Permethrin (Lyclear)	Permethrin (Lyclear)	Permethrin (Lyclear)	Permethrin (Lyclear)	Permethrin (Lyclear), Malathion (Derbac-M)	Permethrin (Lyclear)	Permethrin (Lyclear), Malathion (Derbac-M)	Permethrin (Lyclear)	Permethrin (Lyclear)	Permethrin (Lyclear),	
**Examined residents given oral ivermectin**							1 resident, 1 dose of 7.4mg		*	1 resident, 1 dose of 12mg	
**Home size (examination criteria)**	Small home (examination offered to all)	Large home (as many as possible)	Large home (as many as possible)	Small home (examination offered to all)	Small home (examination offered to all)	Small home (examination offered to all)	Large home (wings affected examined)	Small home (examination offered to all)	Small home (examination offered to all)	Large home (wing affected, access limited)	
**Current residents**	28	57	61	33	34	35	60	36	13	75	**Total**
**Examined residents**	20 (71%)	22 (39%)	22 (36%)	30 (91%)	28 (82%)	27 (77%)	33 (55%)	25 (69%)	13 (100%)	10 (13%)	230
**Examined residents diagnosed with scabies**	2 (10%)	8 (36%)	11(50%)	6 (20%)	2 (7%)	3 (11%)	10 (30%)	6 (24%)	8 (62%)	5 (50%)	61
**Examined residents with crusted scabies**		**		***			1 (10%)			2 (40%)	3
**Residents first diagnosed with scabies at initial visit**	1	4	8	1		1	6	5	4		30
**Residents diagnosed with scabies prior to initial visit**	2	6	3	5	2	2	4	2	4	6	36
**Total no. of residents diagnosed with scabies**	3	10	11	6	2	3	10	7	8	6	66
**No. of residents' time to diagnosis calculated for**	2	7	7	3	2	2	7	6	8	4	48
**Median days to diagnosis (IQR)**	62 (2-122)	8 (7-22)	12 (11-36)	14 (0-105)	31.5 (10-53)	39 (25-53)	20 (2-250)	7.5 (6-30)	307.5 (24.5-663.5)	330.5 (161-746)	22 (7.5-186)

Outbreak characteristics Time to diagnosis (in days) for individual residents with scabies was defined as the time between first awareness of signs or symptoms (earliest report from resident or staff, or RNC records) to date of diagnosis. *Care home V had suffered a prolonged undiagnosed outbreak, in which multiple prescriptions for Lyclear were issued and one resident treated with oral ivermectin despite the RNC never receiving a diagnosis of scabies before the initial clinical visit. The resident prescribed ivermectin died prior to the preliminary visit. **One resident recruited at a preliminary visit had already been diagnosed with crusted scabies by a hospital dermatologist, but had been readmitted into hospital prior to the initial clinical visit and died eight days after diagnosis. ***Care home F included a day centre service where elderly members of the community shared a lounge with residents. A user diagnosed with crusted scabies by other clinicians was not present during any visits and was thus not included in the study.

ID	Age	Scabies diagnosis on initial visit	I Immediate and initiating causes of death	II Other significant conditions contributing to death
**AR7**	90-94	Probable	(Ia) Cardiovascular (Ib) Cardiovascular	Diabetes and Cardiovascular
**BR10**	85-89	No Sign of Scabies	(Ia) Respiratory	Diabetes, Cerebrovascular accident, Cardiovascular
**BR13**	90-94	Probable	(Ia) Dementia	Cardiovascular
**BR24**	85-89	Died before examination – hospital diagnosed crusted scabies	(Ia) Sepsis (Ib) Cardiovascular	Renal failure, Dementia
**ER12**	90-94	Possible	(Ia) Respiratory	
**ER15**	90-94	No sign of scabies	(Ia) Respiratory (Ib) Old Age	(a) Other (b) Other
**ER19**	95-99	No sign of scabies	(Ia) Old Age	Other
**ER21**	85-89	Possible	(Ia) Respiratory (Ib) Respiratory	Dementia
**HR18**	90-94	Probable	(Ia) Cardiovascular	Cardiovascular
**HR22**	85-89	No Sign of Scabies	(Ia) Respiratory	No secondary recorded
**UR11**	85-89	No Sign of Scabies	(Ia) Respiratory (Ib) Respiratory	
**UR15**	95-99	No Sign of Scabies	(Ia) Respiratory	
**UR4**	85-89	No Sign of Scabies	(Ia) Old Age	
**ZR4**	90-94	Definite, crusted grade 2	(Ia) Dementia	Scabies

Causes of death Participant ages and causes of death (declared on death certificates by attending physicians) have been grouped into broad categories to reduce risk of identification of deceased residents. Immediate causes are marked (Ia), initiating causes (Ia) or (Ib), whichever is last.

Search string for 2017 follow-on literature search

Performed in four databases (PubMed, Cinahl, Embase and Web of Science) on 7th January 2017 and repeated on 19th July 2017 using the terms
'(((scabies OR crusted scabies OR sarcoptes scabiei OR scabies mites)) AND (residential home OR care home OR residential facility OR long term care facility OR nursing home)) AND (treatment OR benzyl benzoate OR permethrin OR ivermectin OR malathion OR lindane OR sulfur OR scabicide lotion OR infection control OR washing OR vacuum OR hoover OR cleaning OR carpet OR upholstery OR bedding OR clothes OR isolation OR gloves OR aprons OR care home closure)'.

Sensitivity and use of dermatoscopy for scabies diagnosis

In Discussion we outline other scabies studies as they relate to the sensitivity of dermatoscopy in the elderly. We provide further details and references here to aid the reader. The French hospital based study had 238 participants (mean age, 33y) and was conducted in a dermatology clinic in Paris 2004-2005. Dermatoscopy was reported to have a sensitivity of 91%. [24] The Brazilian study was carried out in 2008 and compared methods for diagnosing scabies in 113 individuals, with a median age of 14y, who were living in 'typical urban slums'. A sensitivity of 83% was reported for dermatoscopy in the cohort.[25] Mounsey et al.[11] report in supplementary material to their review, that only one study concerning 'aged care' facilities stated use of dermatoscopy for the diagnosis of scabies.[WebRef1]

WebRef1 Paasch U and Haustein U. Management of endemic outbreaks of scabies with allethrin, permethrin, and ivermectin. I J Dermatol 2000; 39: 463–470.

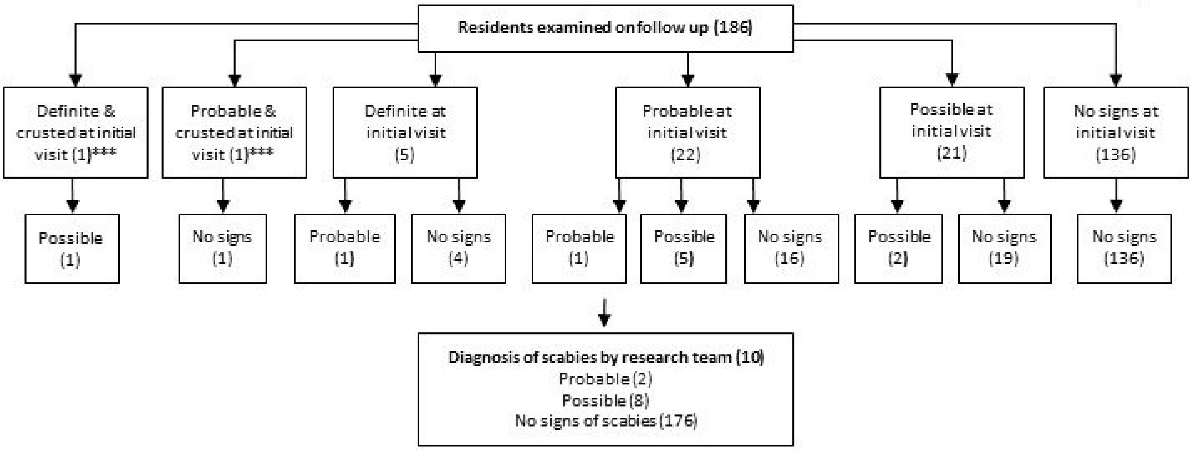

Clinical progression of examined residents at follow-up ***One resident with definite scabies (crusted grade 1) was no longer crusted at follow up, but remained possible. One resident with probable scabies (crusted grade 1) had been treated with oral ivermectin and had no signs at follow up.
